# What is a hospital bed day worth? A contingent valuation study of hospital Chief Executive Officers

**DOI:** 10.1186/s12913-017-2079-5

**Published:** 2017-02-14

**Authors:** Katie Page, Adrain G. Barnett, Nicholas Graves

**Affiliations:** Institute of Health and Biomedical Innovation, Queensland University of Technology, 60 Musk Avenue, Kelvin Grove, Qld 4059 Australia

**Keywords:** Bed days, Valuation, Willingness-to-pay, Cost effectiveness

## Abstract

**Background:**

Decreasing hospital length of stay, and so freeing up hospital beds, represents an important cost saving which is often used in economic evaluations. The savings need to be accurately quantified in order to make optimal health care resource allocation decisions. Traditionally the accounting cost of a bed is used. We argue instead that the economic cost of a bed day is the better value for making resource decisions, and we describe our valuation method and estimations for costing this important resource.

**Methods:**

We performed a contingent valuation using 37 Australian Chief Executive Officers’ (CEOs) willingness to pay (WTP) to release bed days in their hospitals, both generally and using specific cases. We provide a succinct thematic analysis from qualitative interviews post survey completion, which provide insight into the decision making process.

**Results:**

On average CEOs are willing to pay a marginal rate of $216 for a ward bed day and $436 for an Intensive Care Unit (ICU) bed day, with estimates of uncertainty being greater for ICU beds. These estimates are significantly lower (four times for ward beds and seven times for ICU beds) than the traditional accounting costs often used. Key themes to emerge from the interviews include the importance of national funding and targets, and their associated incentive structures, as well as the aversion to discuss bed days as an economic resource.

**Conclusions:**

This study highlights the importance for valuing bed days as an economic resource to inform cost effectiveness models and thus improve hospital decision making and resource allocation. Significantly under or over valuing the resource is very likely to result in sub-optimal decision making. We discuss the importance of recognising the opportunity costs of this resource and highlight areas for future research.

**Electronic supplementary material:**

The online version of this article (doi:10.1186/s12913-017-2079-5) contains supplementary material, which is available to authorized users.

## Background

An effective and efficient hospital-based program will simultaneously both improve patient outcomes and decrease hospital length of stay. Decreasing length of stay and freeing up, or releasing of hospital beds, represents a cost saving. However, the magnitude of this cost saving is difficult to quantify and is likely to depend on many factors. Hospital beds can have two types of value: (1) how much they cost the hospital to run – the accounting cost (referred to as the hotel cost by the WHO [1]), and (2) the value they have in terms of achieving desired outcomes – the economic (or opportunity) cost. Previous cost-effectiveness analyses most often use the accounting cost of some variant of it [2]. This is predominantly because it is an easier value to calculate and to understand, especially by hospital administrators.

The question that arises is to what extent this is the “right” value to be adopting? Arguably, the second value, the economic cost, is the real value we “should” be interested in. This is because the majority of bed costs are fixed and sunk costs, and therefore the “true” value of releasing a hospital bed is better captured by the extent to which it allows one to achieve other outcomes that the hospital desires, such as treat another patient, reduce waiting lists, and ultimately meet economic targets. An accounting value fails to represent economic opportunity costs, rather accounting conventions are used to recover historical expenditures. WTP may be closer to the economic opportunity cost, and that opportunity cost is a basis for decision making, the efficiency of which is the subject of welfare economics. The true economic value of a bed day is probably lower than the full financial accounting cost. The question is, how much lower?

Previous cost-effectiveness research [3] has shown that the dollar value placed on a hospital bed, average cost versus just consumables, is important. Sensitivity analysis around the bed day value demonstrated that it had a significant impact on the overall decisions derived from the cost effectiveness model. Bed day values often drive the cost savings for cost-effectiveness outcomes and thus if we overvalue bed days, by using arbitrary accounting conventions, programs can seem more cost-effective than they really are. For example, bed days saved add to the costs savings side of a cost-effectiveness analysis and thus a reduction in bed day values will lower costs savings, and assuming all other values are unchanged, will decrease the likelihood of an intervention being cost effective. Specifically, it we use a hypothetical value of AUD $1000 per bed day, and intervention “X” results in 100 bed days saved, we have a cost saving of AUD $100,000 (in addition to any other costs savings). If, however, the bed day valuation is lower at AUD $200 we have only a cost saving of AUD $20,000. Therefore, an intervention needs to be less costly or more effective, to offset such a difference. In clear cut cases and with low uncertainty around other model parameters this difference in bed day valuations may not be important. However, arguably there are many cases where there is uncertainty in other parameters and such a fluctuation in values will meaningfully impact on the conclusions to be drawn from the cost effectiveness analysis. The extent to which bed days represent the main portion of the cost savings will directly relate to their overall impact on the outcome. Therefore, there is a need to obtain more accurate measurements for this parameter.

Bed days are a very important cost element in any analysis involving stays in healthcare facilities, therefore an understanding of their value will be important to decision makers trying to maximise the health of patients within a fixed budget. An economic evaluation of beds days could be useful for decision makers because it would help them to maximise patient outcomes and achieve efficiencies in resource use. Longer term it might also serve an educational purpose in that it could enable a shift in current thinking about the resource away from purely financial fixed accounting costs to one where opportunity costs are highlighted to enable sustainable efficiencies.

One can consider two alternative perspectives when thinking about the valuation of hospital beds. First, one could take a broader perspective of the healthcare decision maker who manages waiting lists, and for whom there is a real economic benefit in releasing a bed day for another patient to occupy or for the resource to be used differently. The second perspective is narrower and could be that of a manager working within an Intensive Care Unit (ICU) or hospital. Therefore, who we ask and over what period of time is important.

Previous estimates which have taken a broad perspective include a detailed costing study of an Australian ICU [4] and an economic evaluation which examined spending patterns for Australian public hospital services [5]. Estimates from these studies gave an ICU bed a value of approximately AUD $2600 and a general ward bed a value of AUD $800. These estimates use the average costs over the length of the hospital stay, in a given 12 month period, and do not represent the marginal cost. They include both fixed and variable costs, hence they are unlikely to accurately represent the opportunity cost of this resource.

The alternative perspective considers only the variable cost per bed day. Variable costs are the cash savings that budget holders within the hospital can recover if bed days are not used; they include items such as fluids, dressings and pharmaceuticals. An important issue with the narrow perspective is that costs per bed day decrease over the duration of hospital stay, particularly so for acute beds [6]. We know from previous research that a large proportion (over 80%) of hospital costs are fixed [7] and therefore this variable rate is useful in that it shows what direct savings could be achieved by freeing up a bed. Kahn [8] argues that the cost of an ICU bed is overestimated because of using average costs and not correcting for fixed costs. They estimate that the direct cost savings for the last day of an ICU bed is US$379. In their work they do not measure opportunity costs, which is the value that could be achieved through some alternative use of the resource. Nuti [9] has also demonstrated that some fixed bed costs can actually be considered quasi-fixed on the basis of an alternative use of a bed, the amount of costs recoverable depending on number of beds and the type of intervention. This is exactly the opportunity cost we are interested in measuring. Recent work [10] conducted in Europe using a subset of our methodology has shown also that there is considerable disparity between accounting and economic costs of hospital beds.

For this research we were interested in the broader perspective of the healthcare decision maker reasoning that the Chief Executive Officer (CEO) could, and often does, choose to use this resource for another use when it is released. Therefore, we are interested in the marginal opportunity cost of a hospital bed day. Specifically, we want to know how much CEOs would be willing to pay to free up a bed day in their hospital so they could use it for another purpose, which more accurately represents the choice they actually make. We are also interested in the extent to which this value is comparable with previous cost estimates. To do this we use a contingent valuation method of willingness to pay (WTP) via a direct survey of experts. Whilst we acknowledge this method has some caveats, we chose this approach for two reasons; first because there is no market for such a resource therefore gauging its value through market mechanisms is unrealistic. Second, contingent valuation is a commonly used method in the health domain to elicit values for health gains [11, 12].

This analysis was conducted in the general context of the Australian public health care system and specifically it was performed as part of an overall evaluation of the Australian National Hand Hygiene Initiative (NHHI). Australia’s public hospital system, which provides the majority of acute-care beds, affords free access to hospital care for public patients. It is jointly funded by the Australian federal Government and state/territory governments. However, the public hospitals are run by state and territory governments, and often the CEO of each hospital has discretionary power over how services are run and what investments are made locally. Australian Government funding to the states and territories for public hospitals is made through the National Healthcare Agreement and the National Health Reform Agreement between the Australian Government and the states and territories. One of the main funding models is Activity Based Funding (ABF) which is a way of funding hospitals such that they get paid for the number and mix of patients they treat. If a hospital treats more patients, it receives more funding. However, there is large heterogeneity in funding models and fragmentation of both funding and delivery is the defining characteristic of Australia’s health system [13]. As a result, there are perverse incentives in the system and often this means that decision makers are not incentivised to opt for value-based solutions. Instead doctors and key decision makers in hospitals are often working to maximise revenue and profit. With such power and incentives the value of resources (i.e. beds) could and should be seen as opportunities in which to achieve greater efficiencies.

Within this funding context, the NHHI is a major patient safety programme co-ordinated by Hand Hygiene Australia (HHA) and funded by the Australian Commission for Safety and Quality in Health Care. This program, which commenced in 2009, was designed to improve hand hygiene compliance in every hospital in Australia. It used the WHOs “five moments” program in order to standardise both the training of healthcare workers, and the measurement of compliance, as well as to use a standard definition of *Staphylococcus aureus* bacteraemia infections.

We performed a comprehensive evaluation of both the costs [14, 15] and efficacy [16] of the NHHI, along with a full cost effectiveness evaluation [17]. As part of this evaluation we needed to obtain accurate estimates of the cost savings of the program as determined by the number of hospital beds that were able to be released from preventing infections as well as their value [18]. Because most of the costs of running a hospital are fixed in the short term [7], and the incidence of hospital-acquired infections in Australian hospitals is relatively low, only small cash savings will be made from reducing rates of healthcare associated infections with the real cost of healthcare associated infections being the value of the marginal bed day released to some alternative use. It is of paramount importance to get a good estimate of the economic value of releasing a bed, in this case one linked to a reduction in infections. This was the principal aim of the current study. For decision makers, including hospital administrators, the results from this economic evaluation represent whether the program is good value for money and whether they could achieve efficiencies by better investing their resources in another program.

This paper will discuss how we measured the economic value (opportunity cost) of a bed in the context of the NHHI and discuss the implications this has for evaluating the cost-effectiveness of any program or treatment whose intended outcome is to release hospital beds. We also consider the extent to which this value is useful for assessing cost savings.

## Methods

### Stage 1

There were three stages to this research. First, we conducted three semi-structured interviews with high level decision makers to elicit the important factors likely to drive decision making in this domain. These decision makers included a hospital CEO, a health district level CEO, and a health policy expert in Australia. The interviews were guided by several key questions with the main outputs being (1) a more global understanding of the way in which these types of decision makers think about bed days, and (2) the contextual factors that are likely to influence their decisions concerning how much they value the resource. The factors that emerged from these interviews that were likely to influence decision making are shown in Table 1, along with a summary of the justification/rationale indicated by the decision makers.

**Table 1:** Factors and their rationale for inclusion

Factor	Rationale
Time of year	Infection rates and demand for services in general are impacted upon by seasonal variations.
Bed occupancy	Hospitals generally operate at very high levels of occupancy and to the extent to which this is the case will impact on demand/need for further beds.
Bed type	Ward beds and ICU beds have different demand and different value.
Operating theatre capacity	The extent to which beds can be useful to a hospital at a given point in time is limited by the capacity of the hospital to perform surgery and hence utilise the beds.
Waiting lists	Fluctuations in the external demand for beds/services affect the need for the resource locally.

### Stage 2

A paper-and-pencil contingent valuation survey was developed from these interviews. We used a WTP stated-preference direct survey method with expert decision makers [13]. This was sent to 50 CEOs from the largest public hospitals in Australia covering all states and territories, which was the sample used for the main economic evaluation of the NHHI. These CEOs were pre-determined by the hospitals included in the larger project [15] and do not represent private hospitals. We had a response rate of 75% making a total of 37 surveys.

The survey had three sections. Section 1 was designed to elicit an overall valuation of the importance of beds days, holding other factors constant, and thus this section asked about a general infection control program that frees up to 730 bed days per annum (two beds per day). This number of beds was chosen after consultation with experts on a realistic amount given hospital size and capacity for release.

Section 2 incorporated the different factors shown to be important in the interviews. We designed eight specific scenarios that varied the important factors using a fractional factorial design [19]. This was the most efficient design type for this situation where there were four factors to vary each with two levels (see Additional file 1 for factors and levels). Bed type was valued separately for each scenario. All factors were dichotomous (i.e. high (105%) vs low (85%) bed occupancy). An example scenario is shown in Additional file 2. This design allows one to calculate the main effects without confounding from any 2-way or 3-way interactions between factors.

The third section asked the CEOs to rank the factors for their importance on a seven point Likert scale of importance as well as seeking feedback and general comments on the question about bed day valuations. A oneway ANOVA test was conducted to test for statistical significance between the factors. An example of the full survey can be found in the Additional files 1 and 2.

### Stage 3

A final but very important part of the research was a follow-up telephone call to discuss the rationale and motivations for the decisions. This interview took place in over 80% (*N* = 30) of the completed surveys. This yielded much useful information and thus informed part of the analysis presented below.

## Results

The results are presented in two sections. First, the quantitative results will be described and then a qualitative discussion, taken from the CEO comments in the post-questionnaire interview, will be presented.

### Section 1: Quantitative results

On average the CEOs in our sample had 16 years of experience in hospital management, with an average hospital budget of AUD$475 million per annum. There was considerable variation between hospitals because of differences in hospital size and state budgets.

Holding all factors constant (Section 1 of survey) we found that on average CEOs were willing to pay AU$193,000 per annum (<1% budget) for a general infection control program that saves 730 beds per annum. This equates to AU$264 to free up a bed day in their hospital. 25% of the sample had a WTP of $0. Reasons for this are explored in the discussion. There were missing data for two hospitals. We stress that these WTP estimates do not represent the actual amounts that hospitals paid for the beds in terms of accounting costs but rather the CEOs judgment about how much they would be willing to pay to release this quantity of beds.

We then used the eight scenarios to further explore how this valuation changed, or was predicted by, the various factors. As part of this analysis it became clear that for four scenarios the stated values were very close to zero and this was as a result of the low bed occupancy and hence the unrealistic nature of those scenarios for most hospitals (i.e. the CEOs reported that their hospital was very rarely facing these situations). Therefore, full calculation (as per [19]) of the main effects and interactions for all factors was not deemed appropriate. We therefore selected the four scenarios with high bed occupancy (scenarios 1, 4, 6 and 7) as these were the ones which represented the most common situations facing most hospitals. The four scenarios which were not finally included were similar in all other respects except for the bed occupancy rate (which was low at 85%). We do not believe there is any particular systematic bias introduced by eliminating these four scenarios, however, what it does indicate is that there is probably a threshold value for bed occupancy such that below that threshold CEOs are highly unlikely to be willing to pay anything to free-up beds and over this threshold their WTP increases from zero dependent on others factors as well as the actual bed occupancy rate. The exact threshold is not able to be determined by our study but is a question for further research. The valuations for these scenarios were then taken for both ward and ICU beds and weighted according to the relative amount of time that the CEOs reported their hospital was facing this situation (in weeks). These valuations are shown in Table 2. Missing data were deleted list wise and not imputed.

**Table 2:** Ward and ICU bed day valuations by scenario

Scenario	Ward (AU$)	ICU (AU$)	Weight (~weeks)
S1	237	593	40% (21 weeks)
S4	184	410	20% (10 weeks)
S6	205	472	25% (13 weeks)
S7	224	412	15% (8 weeks)
Mean weighted average	AU$216	AU$436	∑100%

S1: winter, long waiting lists, full OT, high bed occupancy; S4: summer, waiting lists meeting targets, full OT, high bed occupancy; S6: summer, long waiting lists, OT some capacity, high bed occupancy; S7: winter, waiting lists meeting targets, OT some capacity, high bed occupancy

On average CEOs were willing to pay AUD$216 for a ward bed day and AUD$436 for an ICU bed day. To incorporate uncertainty and variability in these estimates we calculated a range based on three standard deviations from the mean which gives AUD$147- AUD$285 for a ward bed and AUD$178-$AUD694 for an ICU bed. These figures were used in our cost-effectiveness model [16] but they are also useful because they show that there is greater uncertainty about the economic value of an ICU bed. This may be because some hospitals have more ICU beds than others, and/or less flexibility for using this resource for other purposes.

The final section asked the CEOs for their ratings of importance of six factors on a seven point Likert scale from 1: Not at all important to 7: Very Important. Table 3 shows the average ratings for the six factors to potentially impact bed day valuations and how important these factors were to the CEOs when making their valuations. The higher the rating the more important the factor. All factors were of moderate importance but “bed occupancy” was the most important factor and “hospital size” the least important factor. A oneway ANOVA showed that mean differences were statistically significant, F (5, 27) = 10.89, *p* < .001, which means that these two factors (hospital size and bed occupancy) are different from the average of the others. The implication of these differences is that bed valuations are more likely to fluctuate with changes in bed occupancy levels than changes in all of the other factors.

**Table 3:** Mean self-report ratings of the importance of the factors

Factor	Mean (SD)
Bed type	4.32 (1.41)
Time of year	4.15 (1.76)
Operating theatre capacity	4.24 (1.69)
Waiting list	4.59 (1.40)
Bed occupancy	5.88 (1.41)*
Hospital size	3.76 (1.89)*

* *p* < .05. (Min 1: Strongly disagree, Max 7: Strongly agree)

### Section 2: Qualitative results

Two main themes emerged from the follow-up interviews with the CEOs. One pertinent factor was the context in which these decisions were being made, specifically the impact of funding and targets on the decision making process.

#### Theme 1: Impact of funding type and targets

In jurisdictions where there is ABF (activity based funding) there is a clear disincentive for producing activity that goes beyond the allocated targets and therefore, where the CEOs sit relative to their targets is often a key consideration in the value they will attach to the resource. If they are already above their targets for a given activity type them the resource will be of no or little value, and this explains the zero dollar valuations for some CEOs. This is demonstrated by one CEO who said: “work activity units (WAUs) are a key driving force” Hospitals get an allowance at start of financial year and we have to work within that”. In all states that had activity based funding all CEOs mentioned the need to consider NEAT (National Emergency Access Target) when deciding on the usefulness of the resource. Specifically, many CEOs used NEAT as an example of a target which was impacting on how they managed their patients through the hospital and thus demonstrating that certain beds are critical at certain times.

However, some CEOs acknowledged that no matter where they were relative to targets they would be able to use the resource to solve other problems in the hospital, even if that meant closing down the beds. In fact, many CEOs were very happy to pay for the beds and then try to offset that cost by closing down wards (in blocks of four). This was mentioned a total of six times by CEOs and some of them indicated they had done this in the past as a way of saving money. Interestingly it has been shown in a Eurpoean context [9] that if the number of beds to be released is below 12 then only limited variable costs can be saved. In the Australian context four beds was considerable a minimum number such that shifting of personnel was possible and costs avoidable.

Overall, it is important to note that the funding structures in different countries and regions will differ considerably and this is likely to have an impact of the final valuations obtained. This research is only generalisable in the Australian public health sector context. In a free market where the CEOs are much less constrained, such as the private hospital sector, then there will likely be more creative use of such a resource, and hence greater variability in the resource value. We were not able to evaluate this difference because the present study only focussed on the public hospital sector.

The second key theme to emerge was the desire to focus on patient quality as a primary outcome and not the amount of bed days saved.

#### Theme 2: Desire to focus on patient quality outcomes and not bed days

The notion of paying for a bed as a resource was viewed negatively by some CEOs/hospital decision makers. Several indicated that they thought there should be a greater focus on patient outcomes relative to the resource use and found it difficult to detach the valuation from this other aspect. This may have affected their responses. In fact about 25% of the CEOs indicated they would not be willing to pay anything for the bed days because they would increase the costs for them in the short term. This suggests that future work could adapt the survey to make it more ecologically valid to the decision making context. However, in spite of this reluctance most were still able to see that the resource did indeed have a value and were able to start thinking about what this might be.

## Discussion

We have demonstrated that CEOs value beds as a resource and are willing to pay for them, but at a rate which is considerably lower than the accounting cost used by hospital administrators. This research represents the first comprehensive attempt to value the opportunity cost of this important resource that informs many cost effectiveness models. However, it was limited in its ability to fully explore the trade-offs that decision makers were making, or indeed were willing to make, (1) because of sample size, and (2) because of constraints associated with the methodology. A more comprehensive analysis could consider using a multi-criteria decision making tool which more explicitly represent the trade-offs between the important factors. This would be useful from a research and hospital management perspective in terms of managing patient flow. Eliciting preferences and decision making thresholds for key decision makers would help people design the system to better utilise the resource. For example, knowing at what exact level of bed occupancy beds become more useful would assist hospitals to manage patient flow.

However, the figures we obtain are surprisingly close to those of Kahn [8] who calculated the direct variable costs of the last days in both ward (US$279) and ICU (US$397) beds. If anything our figures are a magnitude lower and could suggest that the opportunity costs of the resource are sometimes negative after accounting for the direct variable costs. Indeed the incentive structure of pay- per-service and per average length of stay is likely reflected in these estimates. Increased turnover might increase opportunity costs making the resource less valuable. The WTP of zero for some CEOs is likely reflective of this. Some CEOs mentioned buying the beds and closing them down to save money, which does suggest that opportunity costs of extra beds are high and not cost saving in all situations. This concurs with the work of Nuti [9] who showed that beds in multiple of 12 allows re-organisation of existing resources resulting in cost savings, and beds in multiples of 30 could lead to more substantial structural interventions in order to cost save. The exact numbers of “meaningful bed units” is likely to vary per hospital and certainly per health system.

If this is the case then examining the use of beds days saved as a key indicator of the success of hospital based programs is likely be problematic, particularly in the current pay-for-service context in Australia. Other outcomes measures are likely more reflective of the real savings, either in terms of health, satisfaction, or another quality metric. Further exploration of these alternatives is a matter for future research.

Our research demonstrates that WTP for bed days is likely to vary with the healthcare context and even over time depending on current government legislation, specific health care reforms, and the relative position within a funding cycle. This is not acknowledged by using a fixed administrative cost. In our study this variability is somewhat ameliorated by having states which had not yet transitioned to ABF and those who had been using it for several years. A comparison of these states did not yield any major difference in WTP amounts. However, it is prudent not to assume these values are unchanging and fixed. Rather one is advised to either elicit these values in the key decision making group (using a similar process to the one outlined above) or first perform a sensitivity analysis on the rest of the cost effectiveness data to estimate whether this parameter is likely to be of consequence to the overall findings. If so, one can determine the value (or range of values) at which WTP will change the outcome of the decision and then use these to inform decision makers (and they can decide based on their local factors and individual or group preferences).

We acknowledge that the contingent valuation method has limitations. One of the criticisms often levelled at the approach is around the lack of budget constraints in more standard public preference WTP tasks. However, in this instance we asked senior experts who were budget constrained and very explicitly aware of this constraint: we asked them to state their annual budget in at the beginning of the task. Therefore, we maintain that these values are not likely to be highly skewed by the lack of a budget constraint. We also do not provide any form of starting point for the valuation so we avoid starting point bias and anchoring and adjustment.

This task was quite cognitively demanding for respondents because it is not something they typically value, at least explicitly. We tried to ameliorate this by having an in-depth discussion and allowing them time to complete the survey and ask questions before their final valuations were recorded. However, we accept that this might have biased their valuations if they used financial or accounting heuristics in which to make their valuations. It is impossible to eliminate all biases from these estimations using this methodology but we would like to stress that this is a first attempt to gain some useful estimate of the opportunity costs that key decision makers place on a fundamental hospital resource.

Finally, cost effectiveness analysis is all about priority setting – hospital decision makers should choose the most cost effective solutions to maximise the heath of patients within a fixed budget. In order to do so they need to be sure that the inputs into the models around bed day savings are realistic and likely to come to fruition in reality. Using estimates which over represent the cost savings (as is likely currently the case) will result in programs seeming more cost effective than they are, and thus ultimately sub-optimal decision making and priority setting.

## Conclusions

We have shown one way to calculate the opportunity costs for hospital bed days in a sample of Australian hospital CEOs. We highlight that these figures are significantly lower than the costs savings often attributed to hospital beds in cost effectiveness evaluations and discuss the implications of this over estimation. This research is of general significance because it highlights the importance of exploring the assumptions in cost-effectiveness models, the need to make these explicit, and explore their impact in more detail. Whilst this is a specific case related to the value of hospital beds, the broader ramifications are clear. This research also attests to the importance of quantifying the opportunity costs of a resource and not just the accounting value. Both may be useful so it is important to understand the value needed by decision makers for optimal healthcare decision making. More often than not this is the use of a resource for future problem-solving as opposed to how much has been paid. More studies like this one are needed to accurately assess and understand the value placed on key non-market resources in health care by decision makers under various constraints.

## Additional files

Additional file 1: Summary tables. This includes two tables – one showing factors and their levels and the other providing an example scenario with the different levels. (DOCX 76 kb)
Additional file 2: Questionnaire. This file shows the full questionnaire given to one of the participants. (DOCX 361 kb)
