# Supplementary material for: What is a hospital bed day worth? A contingent valuation study of hospital Chief Executive Officers
Source: BMC Health Serv Res. 2017 Feb 14;17:137. doi: 10.1186/s12913-017-2079-5 (PMC5310013; doi:10.1186/s12913-017-2079-5)
Supplement: Additional file 1: — Summary tables. This includes two tables – one showing factors and their levels and the other providing an example scenario with the different levels. (DOCX 76 kb) [file 12913_2017_2079_MOESM1_ESM.docx]

**Additional file 1: Factors and their levels**

| **Factor** | **Low Level** | **High level** |
| --- | --- | --- |
| Time of year | Summer | Winter |
| Bed occupancy | 85% | 105% |
| Waiting list | Waiting list times are acceptable  (meeting targets) | Very long |
| Operating theatre capacity | Some capacity | Full |

**Appendix B: Example Scenario**

| 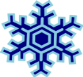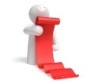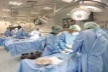 *105%* | It is winter |
| --- | --- |
|  | Waiting lists are very long |
|  | The operating theatre is full |
|  | Bed occupancy is 105% |

Thinking about this specific situation how much would you be **willing to pay** to free up:

**2** **WARD BEDS per day (730 bed days per year)** of your existing capacity?

**1 ICU BED per day (365 bed days per year)** of your existing capacity?
