# Supplementary material for: What is a hospital bed day worth? A contingent valuation study of hospital Chief Executive Officers
Source: BMC Health Serv Res. 2017 Feb 14;17:137. doi: 10.1186/s12913-017-2079-5 (PMC5310013; doi:10.1186/s12913-017-2079-5)
Supplement: Additional file 2: — Questionnaire. This file shows the full questionnaire given to one of the participants. (DOCX 361 kb) [file 12913_2017_2079_MOESM2_ESM.docx]

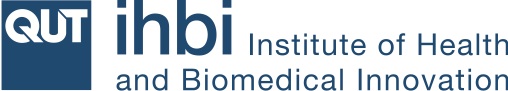


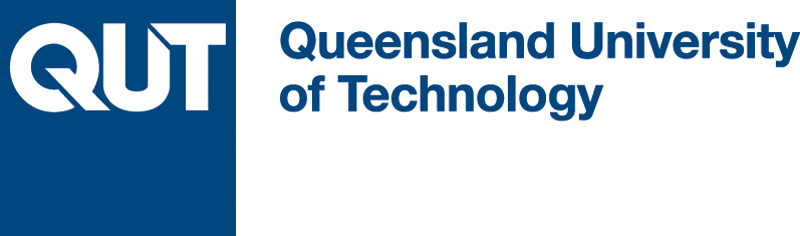


This survey will ask you about how much you value, in monetary terms, the beds in your hospital. It has three (3) sections.

**Section 1:** *Questions about a specific program that releases hospital beds*

**Section 2:** *Questions based on specific scenarios*

**Section 3:** *Summary questions about your responses*

For each of your responses we would really like you to think carefully and weigh up the options before providing your responses. There are eight unique scenarios and each requires that you assess the situation and provide two key responses. In total this survey will take between 15-25 minutes.

We realise there are many factors likely to influence your choice at any given time and we have only isolated a few very important ones. Please focus on the information which is in the scenario and **assume other variables/factors are constant** across the scenarios. There will be a chance to comment at the end.

If you have any questions then please contact either:

Dr Katie Page,

Research Fellow

Institute of Health and Biomedical Innovation

07 3138 0770

[katie.page@qut.edu.au](mailto:katie.page@qut.edu.au)

or

Professor Nick Graves

Professor of Health Economics and Academic Director - Australian Centre for Health Services Innovation (AusHSI)

Institute of Health and Biomedical Innovation

07 3138 6115

[n.graves@qut.edu.au](mailto:n.graves@qut.edu.au)

**Demographics**

What is the name of your hospital? ____________________________________________________________

How many years have you been working in health care management? _____________ **years**

What was your total hospital budget for 2011/2012 financial year? $ _____________________

Imagine a new infection control program which is being marketed to different hospitals in Australia. It is predicted that this new program will reduce the rates of nosocomial infection over the next year and a key benefit will be the freeing up of beds within your existing capacity.

It is anticipated that this new program will release **2 beds per day** for the next year (roughly **730 bed days per annum)** in your hospital.

What is the **maximum amount** you would be willing to pay, from your existing budget, to have this program in your hospital?

$______________________

What percentage of your total financial budget would you release to invest in it?

______________________%

How many **total** beds* do you have in your hospital? _______________________**beds**

How many **acute** beds* do you have in your hospital? **_______________________ beds**

How many **ICU** beds* do you have in your hospital? _______________________**beds**

*** *In the 2011-2012 Financial Year***

**What follows are eight (8) unique scenarios in which each of the four factors differs. We ask that you please consider each scenario carefully.**

## Scenario 1

Imagine your hospital in the following situation:

| 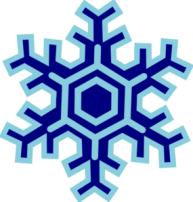 | **It is winter** |
| --- | --- |
| 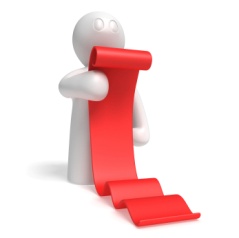 | **Waiting lists are very long** |
| 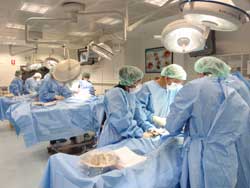 | **The operating theatre is full** |
|  | **Bed occupancy is 105%** |

Thinking about this specific situation how much would you be **willing to pay** to free up:

| **Bed Type** | **Number of Beds** | **Your Valuation** |
| --- | --- | --- |
| WARD BEDS  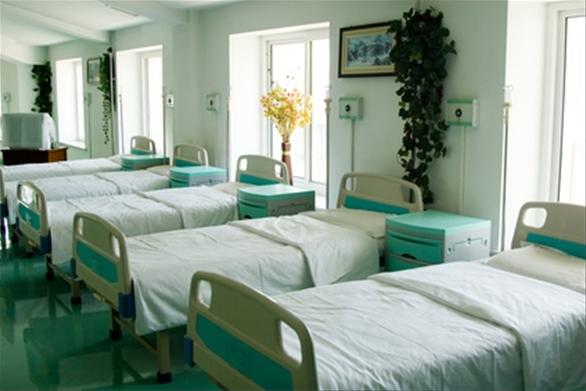 | **2** **WARD BEDS per day (730 bed days per year)** of your existing capacity? | **$** |
| ICU BEDS  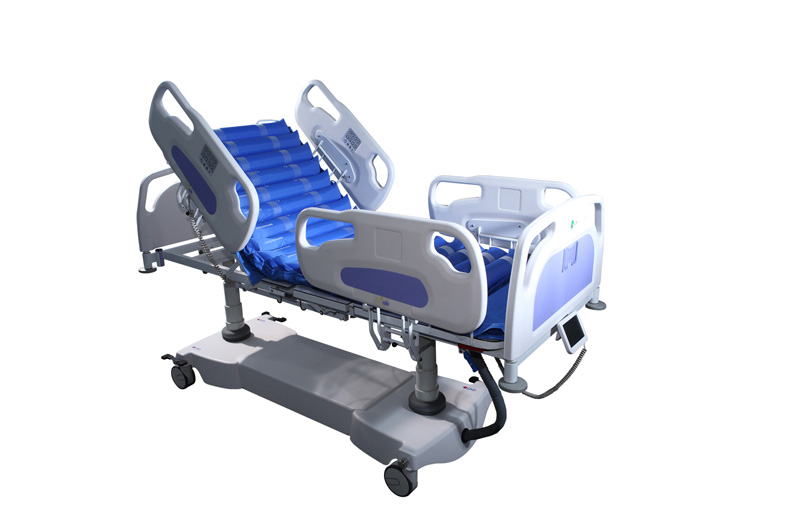 | **1 ICU BED per day (365 bed days per year)** of your existing capacity? | **$** |

Ignoring the time of year (summer or winter), for how many **weeks** of the year do you think your hospital would experience this, or a very similar, situation *(please circle one of the numbers below)*?

| 1 | 2 | 3 | 4 | 5 | 6 | 7 | 8 | 9 | 10 | 11 | 12 | 13 |
| --- | --- | --- | --- | --- | --- | --- | --- | --- | --- | --- | --- | --- |
| 14 | 15 | 16 | 17 | 18 | 19 | 20 | 21 | 22 | 23 | 24 | 25 | 26 |
| 27 | 28 | 29 | 30 | 31 | 32 | 33 | 34 | 35 | 36 | 37 | 38 | 39 |
| 40 | 41 | 42 | 43 | 44 | 45 | 46 | 47 | 48 | 49 | 50 | 51 | 52 |

## Scenario 2

Imagine your hospital in the following situation:

| 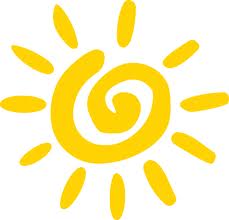 | **It is summer** |
| --- | --- |
| 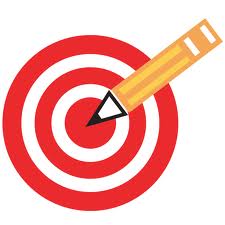 | **Waiting list times are acceptable**  **(meeting targets)** |
| 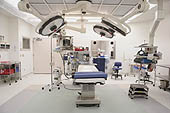 | **The operating theatre has some capacity** |
|  | **Bed occupancy is 85%** |

Thinking about this specific situation how much would you be **willing to pay** to free up:

| **Bed Type** | **Number of Beds** | **Your Valuation** |
| --- | --- | --- |
| WARD BEDS  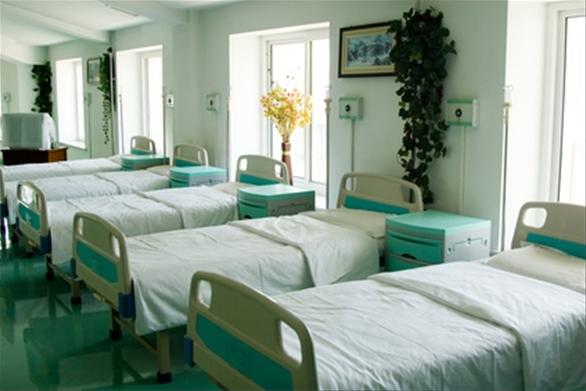 | **2** **WARD BEDS per day (730 bed days per year)** of your existing capacity? | **$** |
| ICU BEDS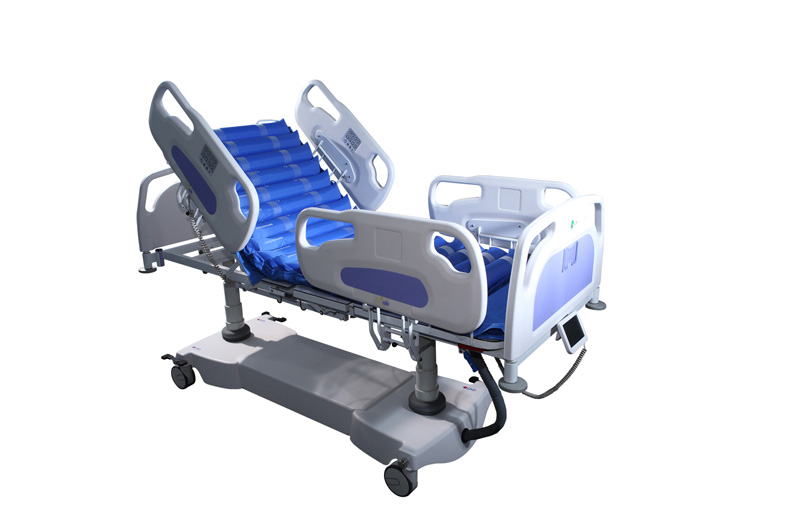 | **1 ICU BED per day (365 bed days per year)** of your existing capacity? | **$** |

Ignoring the time of year (summer or winter), for how many **weeks** of the year do you think your hospital would experience this, or a very similar, situation *(please circle one of the numbers below)*?

| 1 | 2 | 3 | 4 | 5 | 6 | 7 | 8 | 9 | 10 | 11 | 12 | 13 |
| --- | --- | --- | --- | --- | --- | --- | --- | --- | --- | --- | --- | --- |
| 14 | 15 | 16 | 17 | 18 | 19 | 20 | 21 | 22 | 23 | 24 | 25 | 26 |
| 27 | 28 | 29 | 30 | 31 | 32 | 33 | 34 | 35 | 36 | 37 | 38 | 39 |
| 40 | 41 | 42 | 43 | 44 | 45 | 46 | 47 | 48 | 49 | 50 | 51 | 52 |

## Scenario 3

Imagine your hospital in the following situation:

| 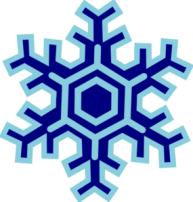 | **It is winter** |
| --- | --- |
| 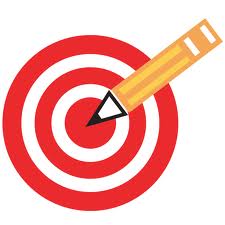 | **Waiting list times are acceptable**  **(meeting targets)** |
| 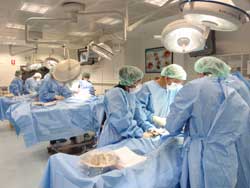 | **The operating theatre is full** |
|  | **Bed occupancy is 85%** |

Thinking about this specific situation how much would you be **willing to pay** to free up:

| **Bed Type** | **Number of Beds** | **Your Valuation** |
| --- | --- | --- |
| WARD BEDS  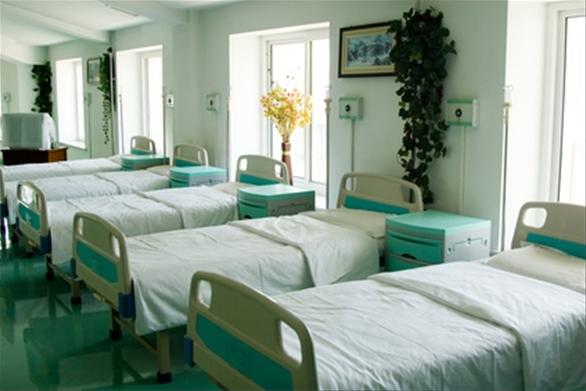 | **2** **WARD BEDS per day (730 bed days per year)** of your existing capacity? | **$** |
| ICU BEDS  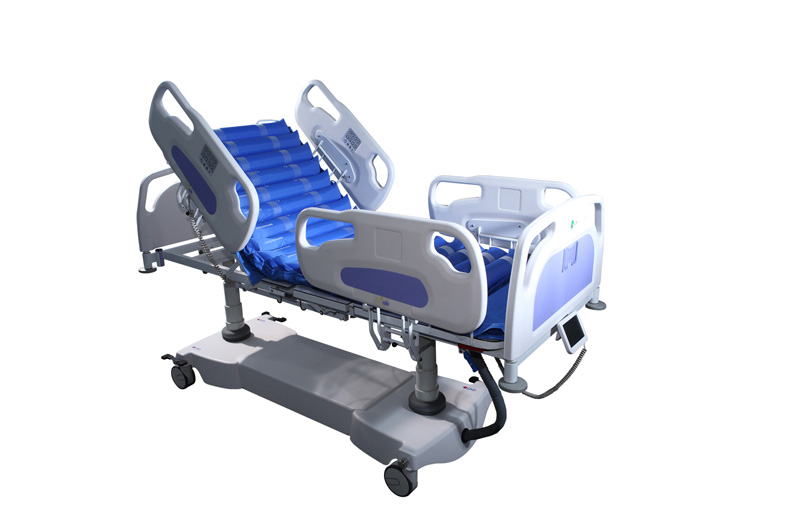 | **1 ICU BED per day (365 bed days per year)** of your existing capacity? | **$** |

Ignoring the time of year (summer or winter), for how many **weeks** of the year do you think your hospital would experience this, or a very similar, situation *(please circle one of the numbers below)*?

| 1 | 2 | 3 | 4 | 5 | 6 | 7 | 8 | 9 | 10 | 11 | 12 | 13 |
| --- | --- | --- | --- | --- | --- | --- | --- | --- | --- | --- | --- | --- |
| 14 | 15 | 16 | 17 | 18 | 19 | 20 | 21 | 22 | 23 | 24 | 25 | 26 |
| 27 | 28 | 29 | 30 | 31 | 32 | 33 | 34 | 35 | 36 | 37 | 38 | 39 |
| 40 | 41 | 42 | 43 | 44 | 45 | 46 | 47 | 48 | 49 | 50 | 51 | 52 |

## Scenario 4

Imagine your hospital in the following situation:

| 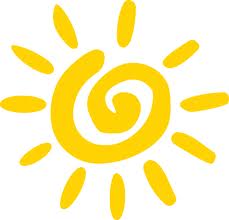 | **It is summer** |
| --- | --- |
| 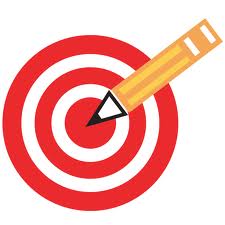 | **Waiting list times are acceptable**  **(meeting targets)** |
| 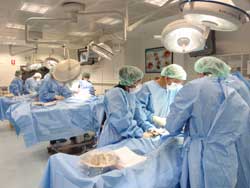 | **The operating theatre is full** |
|  | **Bed occupancy is 105%** |

Thinking about this specific situation how much would you be **willing to pay** to free up:

| **Bed Type** | **Number of Beds** | **Your Valuation** |
| --- | --- | --- |
| WARD BEDS  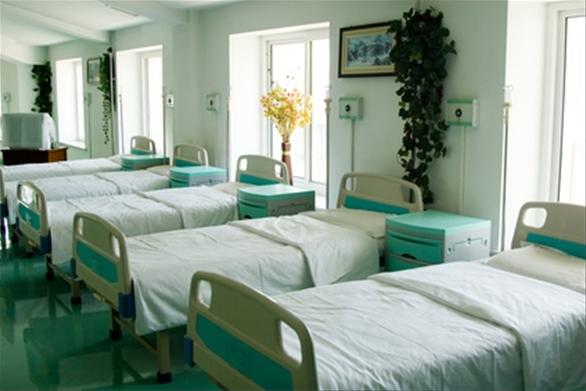 | **2** **WARD BEDS per day (730 bed days per year)** of your existing capacity? | **$** |
| ICU BEDS  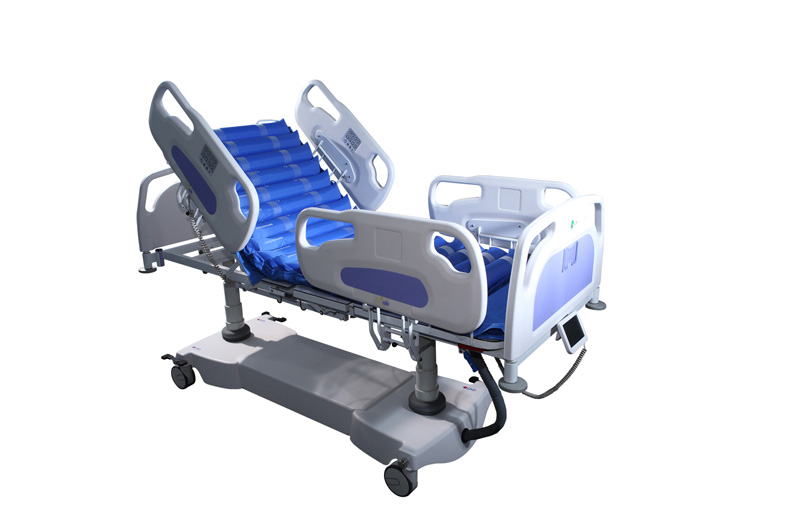 | **1 ICU BED per day (365 bed days per year)** of your existing capacity? | **$** |

Ignoring the time of year (summer or winter), for how many **weeks** of the year do you think your hospital would experience this, or a very similar, situation *(please circle one of the numbers below)*?

| 1 | 2 | 3 | 4 | 5 | 6 | 7 | 8 | 9 | 10 | 11 | 12 | 13 |
| --- | --- | --- | --- | --- | --- | --- | --- | --- | --- | --- | --- | --- |
| 14 | 15 | 16 | 17 | 18 | 19 | 20 | 21 | 22 | 23 | 24 | 25 | 26 |
| 27 | 28 | 29 | 30 | 31 | 32 | 33 | 34 | 35 | 36 | 37 | 38 | 39 |
| 40 | 41 | 42 | 43 | 44 | 45 | 46 | 47 | 48 | 49 | 50 | 51 | 52 |

## Scenario 5

Imagine your hospital in the following situation:

| 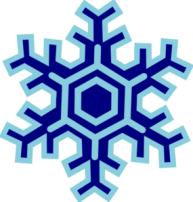 | **It is winter** |
| --- | --- |
| 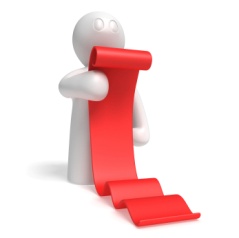 | **Waiting list times are very long** |
| 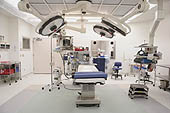 | **The operating theatre has some capacity** |
|  | **Bed occupancy is 85%** |

Thinking about this specific situation how much would you be **willing to pay** to free up:

| **Bed Type** | **Number of Beds** | **Your Valuation** |
| --- | --- | --- |
| WARD BEDS  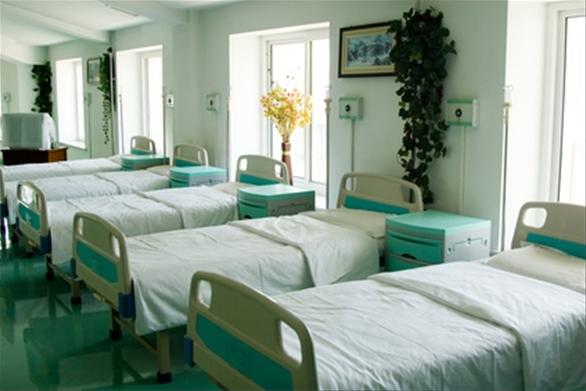 | **2** **WARD BEDS per day (730 bed days per year)** of your existing capacity? | **$** |
| ICU BEDS  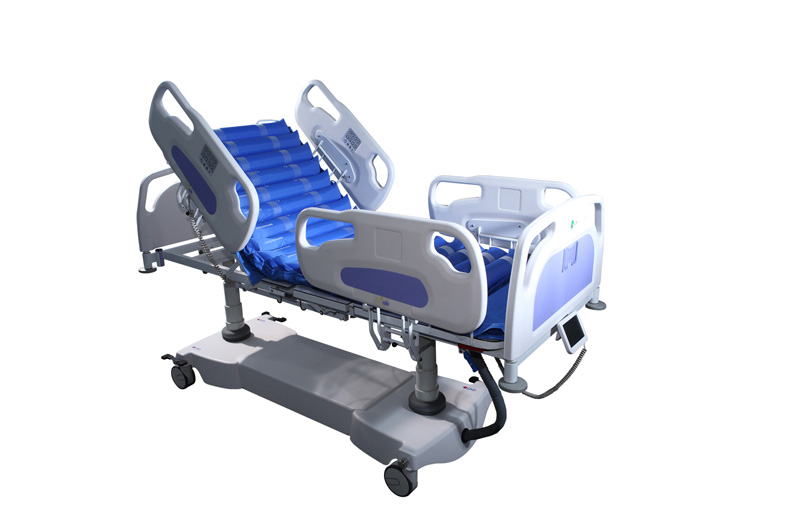 | **1 ICU BED per day (365 bed days per year)** of your existing capacity? | **$** |

Ignoring the time of year (summer or winter), for how many **weeks** of the year do you think your hospital would experience this, or a very similar, situation *(please circle one of the numbers below)*?

| 1 | 2 | 3 | 4 | 5 | 6 | 7 | 8 | 9 | 10 | 11 | 12 | 13 |
| --- | --- | --- | --- | --- | --- | --- | --- | --- | --- | --- | --- | --- |
| 14 | 15 | 16 | 17 | 18 | 19 | 20 | 21 | 22 | 23 | 24 | 25 | 26 |
| 27 | 28 | 29 | 30 | 31 | 32 | 33 | 34 | 35 | 36 | 37 | 38 | 39 |
| 40 | 41 | 42 | 43 | 44 | 45 | 46 | 47 | 48 | 49 | 50 | 51 | 52 |

## Scenario 6

Imagine your hospital in the following situation:

| 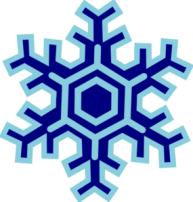 | **It is winter** |
| --- | --- |
| 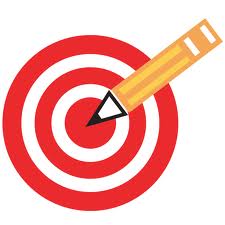 | **Waiting list times are acceptable**  **(meeting targets)** |
| 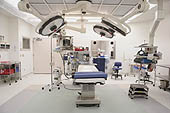 | **The operating theatre has some capacity** |
|  | **Bed occupancy is 105%** |

Thinking about this specific situation how much would you be **willing to pay** to free up:

| **Bed Type** | **Number of Beds** | **Your Valuation** |
| --- | --- | --- |
| WARD BEDS  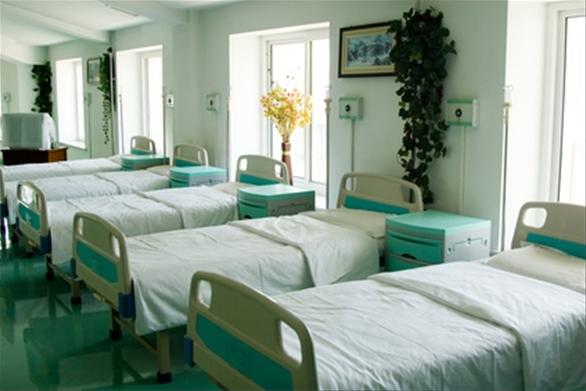 | **2** **WARD BEDS per day (730 bed days per year)** of your existing capacity? | **$** |
| ICU BEDS  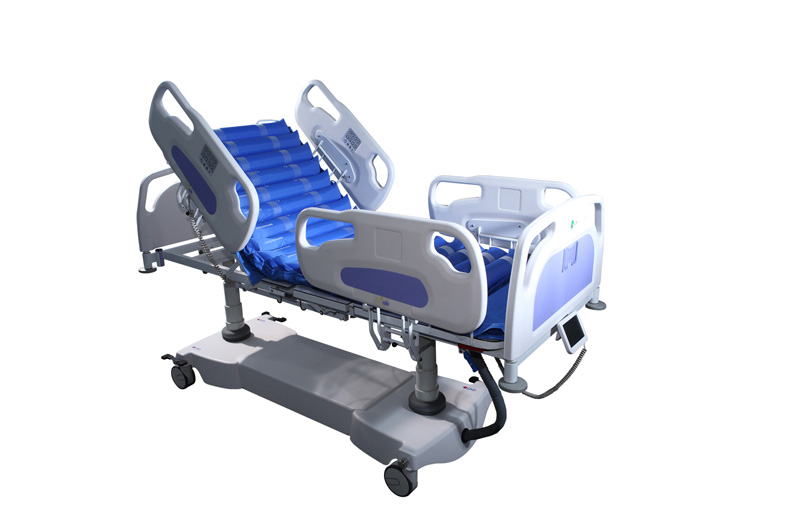 | **1 ICU BED per day (365 bed days per year)** of your existing capacity? | **$** |

Ignoring the time of year (summer or winter), for how many **weeks** of the year do you think your hospital would experience this, or a very similar, situation *(please circle one of the numbers below)*?

| 1 | 2 | 3 | 4 | 5 | 6 | 7 | 8 | 9 | 10 | 11 | 12 | 13 |
| --- | --- | --- | --- | --- | --- | --- | --- | --- | --- | --- | --- | --- |
| 14 | 15 | 16 | 17 | 18 | 19 | 20 | 21 | 22 | 23 | 24 | 25 | 26 |
| 27 | 28 | 29 | 30 | 31 | 32 | 33 | 34 | 35 | 36 | 37 | 38 | 39 |
| 40 | 41 | 42 | 43 | 44 | 45 | 46 | 47 | 48 | 49 | 50 | 51 | 52 |

**Scenario 7**

Imagine your hospital in the following situation:

| 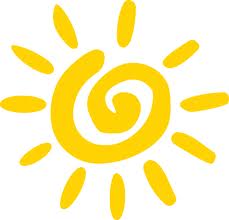 | **It is summer** |
| --- | --- |
| 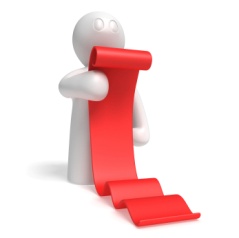 | **Waiting list times are very long** |
| 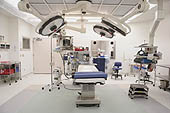 | **The operating theatre has some capacity** |
|  | **Bed occupancy is 105%** |

Thinking about this specific situation how much would you be **willing to pay** to free up:

| **Bed Type** | **Number of Beds** | **Your Valuation** |
| --- | --- | --- |
| WARD BEDS  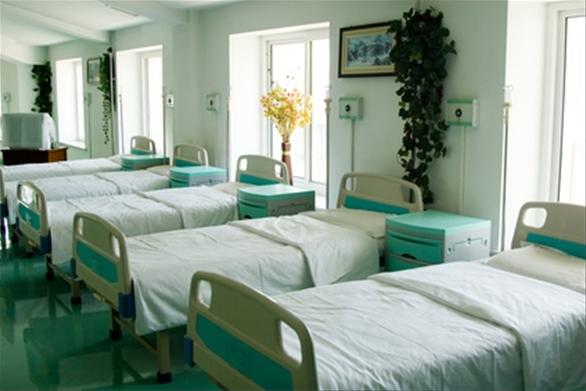 | **2** **WARD BEDS per day (730 bed days per year)** of your existing capacity? | **$** |
| ICU BEDS  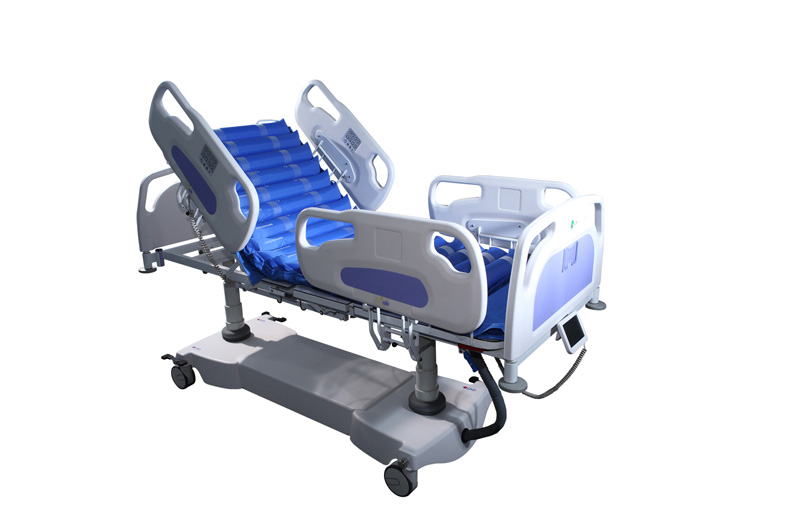 | **1 ICU BED per day (365 bed days per year)** of your existing capacity? | **$** |

Ignoring the time of year (summer or winter), for how many **weeks** of the year do you think your hospital would experience this, or a very similar, situation *(please circle one of the numbers below)*?

| 1 | 2 | 3 | 4 | 5 | 6 | 7 | 8 | 9 | 10 | 11 | 12 | 13 |
| --- | --- | --- | --- | --- | --- | --- | --- | --- | --- | --- | --- | --- |
| 14 | 15 | 16 | 17 | 18 | 19 | 20 | 21 | 22 | 23 | 24 | 25 | 26 |
| 27 | 28 | 29 | 30 | 31 | 32 | 33 | 34 | 35 | 36 | 37 | 38 | 39 |
| 40 | 41 | 42 | 43 | 44 | 45 | 46 | 47 | 48 | 49 | 50 | 51 | 52 |

## Scenario 8

Imagine your hospital in the following situation:

| 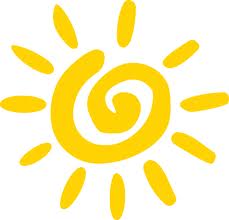 | **It is summer** |
| --- | --- |
| 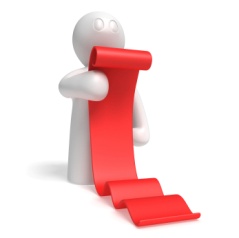 | **Waiting lists are very long** |
| 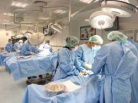 | **The operating theatre is full** |
|  | **Bed occupancy is 85%** |

Thinking about this specific situation how much would you be **willing to pay** to free up:

| **Bed Type** | **Number of Beds** | **Your Valuation** |
| --- | --- | --- |
| WARD BEDS  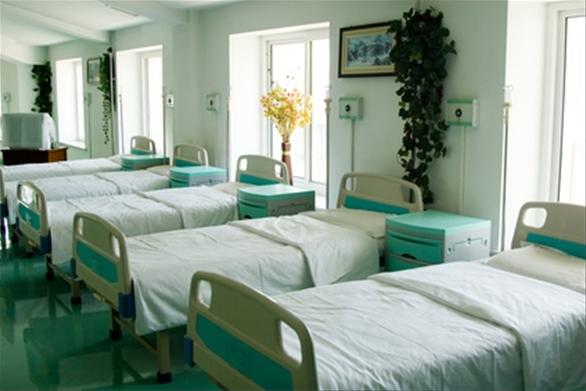 | **2** **WARD BEDS per day (730 bed days per year)** of your existing capacity? | **$** |
| ICU BEDS  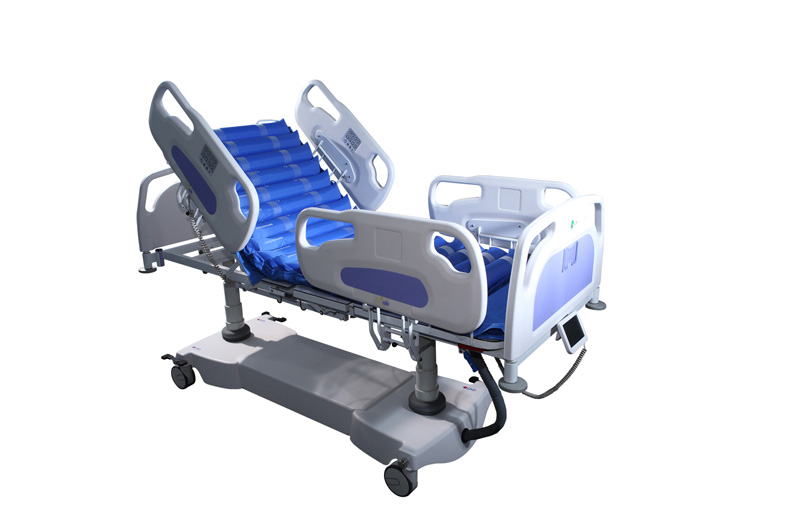 | **1 ICU BED per day (365 bed days per year)** of your existing capacity? | **$** |

Ignoring the time of year (summer or winter), for how many **weeks** of the year do you think your hospital would experience this, or a very similar, situation *(please circle one of the numbers below)*?

| 1 | 2 | 3 | 4 | 5 | 6 | 7 | 8 | 9 | 10 | 11 | 12 | 13 |
| --- | --- | --- | --- | --- | --- | --- | --- | --- | --- | --- | --- | --- |
| 14 | 15 | 16 | 17 | 18 | 19 | 20 | 21 | 22 | 23 | 24 | 25 | 26 |
| 27 | 28 | 29 | 30 | 31 | 32 | 33 | 34 | 35 | 36 | 37 | 38 | 39 |
| 40 | 41 | 42 | 43 | 44 | 45 | 46 | 47 | 48 | 49 | 50 | 51 | 52 |

**Could you please rate on the following 7 point scale how important the five factors were in your valuation of bed days:**

| **1** | **2** | **3** | **4** | **5** | **6** | **7** |
| --- | --- | --- | --- | --- | --- | --- |
| **Not at all Important** | **Slightly Important** | **Somewhat Important** | **Moderately Important** | **Important** | **Very Important** | **Extremely Important** |

| **Factor** | **Importance** | | | | | | |
| --- | --- | --- | --- | --- | --- | --- | --- |
| Bed Type | *1* | *2* | *3* | *4* | *5* | *6* | *7* |
| Time of Year | *1* | *2* | *3* | *4* | *5* | *6* | *7* |
| Operating Theatre Capacity | *1* | *2* | *3* | *4* | *5* | *6* | *7* |
| Waiting List characteristics | *1* | *2* | *3* | *4* | *5* | *6* | *7* |
| Bed Occupancy | *1* | *2* | *3* | *4* | *5* | *6* | *7* |
| Your Hospital Size | *1* | *2* | *3* | *4* | *5* | *6* | *7* |

What factor is the **most important** for determining how much you are willing to pay to gain extra bed days? Why?

|  |
| --- |

Are there any other factors you think influence bed valuations?

|  |
| --- |

**Thank you very much for your time and your responses. They are most valuable and will be a critical part of our evaluation of the National Hand Hygiene Program.**
